# A prospective cohort study of prodromal Alzheimer’s disease: Prospective Imaging Study of Ageing: Genes, Brain and Behaviour (PISA)

**DOI:** 10.1016/j.nicl.2020.102527

**Published:** 2020-12-08

**Authors:** Michelle K. Lupton, Gail A. Robinson, Robert J. Adam, Stephen Rose, Gerard J. Byrne, Olivier Salvado, Nancy A. Pachana, Osvaldo P. Almeida, Kerrie McAloney, Scott D Gordon, Parnesh Raniga, Amir Fazlollahi, Ying Xia, Amelia Ceslis, Saurabh Sonkusare, Qing Zhang, Mahnoosh Kholghi, Mohan Karunanithi, Philip E Mosley, Jinglei Lv, Léonie Borne, Jessica Adsett, Natalie Garden, Jurgen Fripp, Nicholas G. Martin, Christine C Guo, Michael Breakspear

**Affiliations:** a QIMR Berghofer Medical Research Institute, Brisbane, Australia; b School of Psychology, The University of Queensland, St. Lucia, Brisbane, Australia; c Queensland Brain Institute, The University of Queensland, St. Lucia, Brisbane, Australia; d Centre for Clinical Research (UQCCR), The University of Queensland, Brisbane, Australia; e Royal Brisbane and Women's Hospital Mental Health Services, University of Queensland, Brisbane, Australia; f Faculty of Medicine, University of Queensland, Brisbane, Australia; g CSIRO Health and Biosecurity, Australian E-Health Research Centre, Brisbane, Australia; h Medical School, University of Western Australia, Perth, Australia; i WA Centre for Health and Ageing of the University of Western Australia, Australia; j The University of Newcastle, Newcastle, Australia; k Neurosciences Queensland, Brisbane, Queensland, Australia; l Sydney Imaging & School of Biomedical Engineering, The University of Sydney, Sydney, Australia

**Keywords:** Alzheimer’s disease, Neuroimaging, Neuropsychology, Genetic risk prediction, Protocol, At risk cohort

## Abstract

This prospective cohort study, “Prospective Imaging Study of Ageing: Genes, Brain and Behaviour” (PISA) seeks to characterise the phenotype and natural history of healthy adult Australians at high future risk of Alzheimer’s disease (AD). In particular, we are recruiting midlife and older Australians with high and low genetic risk of dementia to discover biological markers of early neuropathology, identify modifiable risk factors, and establish the very earliest phenotypic and neuronal signs of disease onset. PISA utilises genetic prediction to recruit and enrich a prospective cohort and follow them longitudinally. Online surveys and cognitive testing are used to characterise an Australia-wide sample currently totalling over 3800 participants. Participants from a defined at-risk cohort and positive controls (clinical cohort of patients with mild cognitive impairment or early AD) are invited for onsite visits for detailed functional, structural and molecular neuroimaging, lifestyle monitoring, detailed neurocognitive testing, plus blood sample donation. This paper describes recruitment of the PISA cohort, study methodology and baseline demographics.

## Highlights

• Detailed protocol of the Prospective Imaging Study of Ageing (PISA) Study.
• Genetic risk prediction to identify those at differing risk of Alzheimer’s disease.
• Longitudinal cohort for the study of precursors and lifestyle risk factors.
• Use of online surveys and cognitive testing for large scale phenotyping.
• Functional, structural and molecular neuroimaging with neurocognitive testing.

## 1 Introduction

The neurodegenerative process underlying dementia due to Alzheimer’s disease (AD) spans several decades (Morris, 2005). Though the symptomatic burden of dementia typically occurs late in life, it is preceded by a long preclinical phase, characterized by the pernicious accumulation of neuropathology in the brain (Chiti and Dobson, 2006, Villemagne et al., 2013). This preclinical process is believed to commence decades prior to the establishment of functional decline and macroscopic brain atrophy (Dubois et al., 2016). Subtle, largely undetected changes in mood, anxiety and behaviour may accompany this process. Current therapeutic trials have usually targeted those with established AD and therefore, perhaps irreversible neurodegeneration. It is possible that earlier intervention with disease-modifying therapy may increase the chance of halting or averting neurodegenerative processes, and the ensuing burden upon the individual and society. This therapeutic window can only be identified with large-scale studies of those individuals at future risk of AD, but who currently lack substantial incipient neuropathology. The focus, therefore, of much ongoing dementia research is to elucidate early pathological changes, and to identify markers capable of predicting disease progression.

High risk cohorts for AD have typically been identified using neuropsychological tests in older adults, often recruited from “memory clinics” and with cognitive performance threshold abnormal for their age (mild cognitive impairment, MCI) (Gauthier et al., 2006, Winblad et al., 2004). However, longitudinal studies of MCI cohorts are beset by several limitations. First, up to two thirds of people meeting criteria for MCI do not convert to AD within the typical time frame of a longitudinal study (Ganguli et al., 2004, Ravaglia et al., 2006). Second, those who do convert may already possess a considerable burden of AD pathology (Iturria-Medina et al., 2017). Furthermore, the cross-sectional MCI criteria fails to capture the temporal nature of cognitive decline, and lack precision because the estimated baseline performance of individuals varies greatly. Moreover, many people who do develop AD do not pass through, or present with the classic amnestic profile of MCI: Early disturbances in other domains, such as language, visuospatial and executive functions are common (Lam et al., 2013). Alternative strategies, which leverage the potential of genetics and neurobiology, are needed.

The ε4 allele of Apolipoprotein E (*APOE*) is the strongest known genetic risk factor for the common, “sporadic” (also known as late onset) AD (Saunders et al., 1993, Strittmatter et al., 1993). Large scale GWAS meta-analyses have identified at least 24 additional AD genetic risk variants (Harold et al., 2009, Hollingworth et al., 2011;Kunkle et al., 2019, Lambert et al., 2009, Lambert et al. 2013, Naj et al., 2011). While these common genetic variants individually account for a small variance of the disease risk, their combination, as estimated by a polygenic risk score (PRS), explains a substantial amount of the heritability. A high prediction accuracy (~87% of the area under the receiver operation curve, AUC) can be achieved by a prediction model including *APOE* genotype and PRS (containing GWAS association SNPs with *p* value < 0.5). The PRS adds significant predictive value over *APOE* alone (Escott-Price et al., 2015). AD PRS, with the *APOE* region excluded (PRS-no *APOE*), is a robust predictor of age of AD onset, improving the ascertainment of age of AD onset among *APOE* ε3/3 individuals by >10 years between the lowest and highest risk deciles (Desikan et al., 2017). Work from our own group has shown that AD PRS-no *APOE* is associated with reduced hippocampal volume in healthy older adults and those with mild cognitive impairment (MCI) (Lupton et al., 2016). The *APOE* genotype and PRS-no *APOE* both hold an association with longitudinal cognitive decline, structural MRI measures (e.g. hippocampal complex cortical thickness), and radioisotope imaging or cerebrospinal fluid identified amyloid and total tau positivity (Harrison et al., 2016, Tan et al., 2017a, Tan et al., 2017b).

Polygenic risk prediction therefore offers a powerful avenue to identify individuals at high risk of AD. Onto this familial risk is added a host of potentially modifiable risk factors, including hearing loss (Loughrey et al., 2018), insomnia (Xu et al., 2020), cardiovascular (McGrath et al., 2017) and metabolic (Kivimaki et al., 2018) health, traumatic brain injury and lifestyle factors, such as diet (Opie et al., 2013), social isolation, sedentary behaviour (Sabia et al., 2017) and smoking [for review, see (Livingston et al., 2020)]. Studying healthy midlife and older adults at high genetic risk of future AD therefore offers a unique opportunity across a number of domains: to understand the neuropathological processes from their putative onset; the influence of modifiable risk factors on the evolution of these changes; the emergence of comorbid symptoms (such as low mood and anxiety); the variable onset of cognitive changes during conversion from high risk to AD; and the relationship between molecular, structural and functional brain changes. To do so requires ascertainment of such a cohort and detailed phenotypic characterization at regular periods, using imaging, neurocognitive assessment, genetic and biochemical characterization, lifestyle and mental health factors.

Here, we use existing cohorts with available GWAS data to establish a longitudinal cohort with elevated risk for AD to study precursors and lifestyle risk factors for AD. Our *Prospective Imaging Study of Ageing: Genes, Brain and Behaviour (PISA)* comprises the following overarching objectives:

(1) Identify healthy middle-aged and older Australians at high risk of dementia;
(2) Discover biological markers and phenotypic characteristics of early neuropathology;
(3) Identify modifiable risk factors;
(4) Establish a cohort of preclinical patients for future clinical trials.

Several specific imaging innovations were developed for our study. First, we adapted several advanced HCP protocols for the PRISMA platform, using a combination of multiband, multi-echo and multishell dMRI, fMRI, sMRI and QSM sequences. Second, we did this in a manner that was optimized for a time-limited session suitable for older, clinical populations, breaking acquisitions into brief time blocks were possible. Third, we acquire simultaneous MRI and PET (amyloid) images, using the integrated mMR platform to acquire resting state EPI and ASL sequences that serve both as motion correction data for PET image and for resting state BOLD and ASL sequences in their own right.

## 2 Material and methods

### 2.1 Study design

**Fig. 1:**
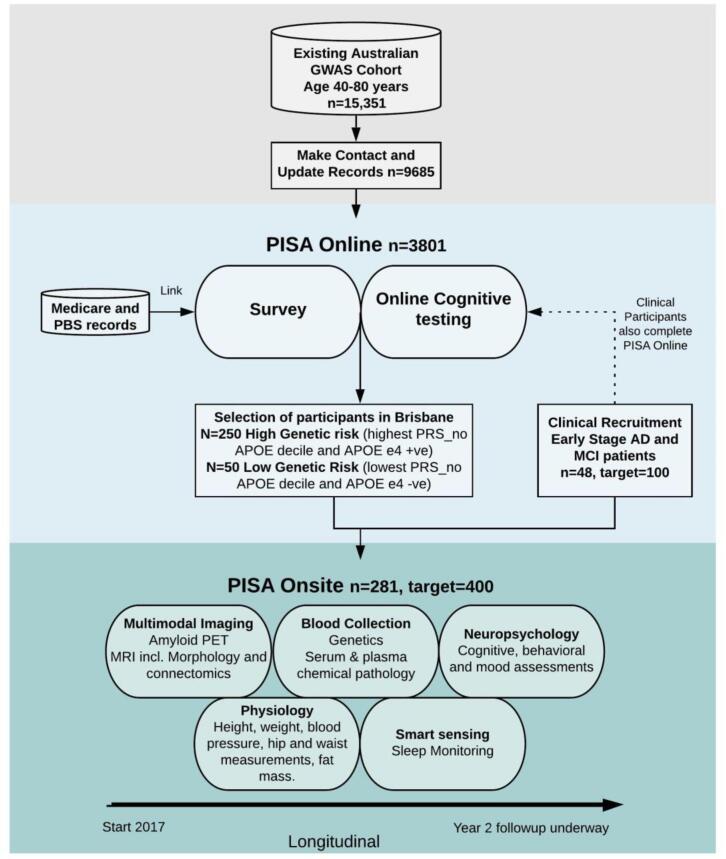
Overview of the PISA study. Sample sizes are current as of 21/08/2020.

PISA is a prospective cohort of healthy Australians at mid to late adulthood, who occupy the two tails of the genetic risk spectrum for late-onset AD. PISA participants derive from extensive in-house cohorts drawn from longitudinal studies of Australian twins and their families conducted over four decades with available genome wide genotyping data to enable a genetically enriched cohort (Fig. 1). The PISA study protocol has approval from the Human Research Ethics Committees (HREC) of QIMR Berghofer Medical Research Institute and the University of Queensland.

### 2.2 PISA online

#### 2.2.1 Online survey

The sample recruitment pool (N = 15,351) comprises adult twins, their spouses, and first-degree relatives of twins and spouses who, over previous decades have volunteered for studies on risk factors or biomarkers for physical or psychiatric conditions and have previously been genome-wide genotyped (Benyamin et al., 2009, Heath et al., 2011). Volunteers have previously given informed consent to be contacted to take part in additional studies. These previous studies span a period of up to 40 years. Postal, telephone or email re-contact of all research participants in the age range of 40–80 years old (dob: years 1946–1986), was undertaken in order to introduce the study and update contact details. Where contact was lost, we endeavoured to obtain participant’s most recent residential address by accessing electoral roll information (available for public inspection at Australian Electoral Commission offices). Currently 9685 (63%) participant records have been successfully updated with attempts at contact ongoing.

All participants who respond to this initial contact are invited to complete an online survey providing a global assessment of lifestyle, cognitive and behavioural function. A comprehensive online survey using Qualtrics Survey software (Qualtrics, Provo, UT, USA) allows participants to complete the survey from home (Table 1). Participants are invited by email with a link to the survey. The survey begins with an information and consent page followed by a core module that captures the central dataset (Table 1 B). We also ask for permission to link participant data with MBS (Medicare Benefits Scheme) and PBS (Pharmaceutical Benefits Scheme) records. Australia has a universal health care system and this allows us to access all government subsidized medical care and prescriptions from the previous 4.5 years. Once the core module is complete, the remaining survey is made up of ten additional modules (Table 1 A), which can be completed at any time in any order, with the typical time required for completion provided on the menu screen. The survey includes over 30 validated instruments (full details are listed in Appendix A). To foster collaboration, the questions were harmonized with data acquired in other national and international dementia and elderly cohorts (including the Older Australian Twin Study (OATS) (Sachdev et al., 2009), the Healthy Brain Aging (HBA) Clinic cohort eHealth survey (LaMonica et al., 2017), the Australian Imaging Biomarkers and Lifestyle (AIBL) study (Ellis et al., 2009), the European Medical Information Framework for AD (EMIF-AD) PreclinAD study (Konijnenberg et al., 2018) and the Brain Health Registry (Weiner et al., 2018). There is currently no time limit for the completion of the online survey and participation follow-up is currently ongoing. In addition, upon request or when email contact has not been established, paper copies of the core module and module 3 (medical history) are mailed to participants for completion. The participant can then complete the remaining modules online if they are able and wish to.

**Table 1:** Summary of PISA online survey.

A. PISA Online Survey Modules	Average time to complete (median m:s)
1. Core Module (see B. below)	26:00
2. Memory & Cognition	8:31
3. Medical History	12:27
4. Personal Wellbeing	12:02
5. Lifestyle	15:17
6. Personality	15:24
7. Life Events	15:40
8. Feelings & Emotions	13:00
9. Physical Health	17:04
10. Women’s Health	5:50
11. Pain	3:37

B. PISA Online Survey Core Module Items	

Key demographics	
Education and occupation	
Extended family	
Biological family medical history	
General memory and health questions	
Alcohol/substance abuse & smoking status	
Patient Health Questionnaire (PHQ-9)	
Generalised Anxiety Disorder (GAD-7)	
Medications checklist	
Active Australia Survey	
Brief personal medical history (PISA exclusion criteria)	

A. Names of each of the 11 modules and the time taken for completion.

B. Sections included in the core module.

Full details including instruments used and references are listed in Appendix A.

#### 2.2.2 Online cognitive testing

All participants who complete the survey are invited to undertake online cognitive testing (Table 2). Participants are first invited to complete the online cognitive assessments via the Cambridge Brain Sciences (CBS) platform (Corbett et al., 2015) approximately two to three months after completion of the online survey. Participants are then approached to complete the Cogstate (Mielke et al., 2015) and Emotion Recognition (Kessels et al., 2014) tests. A second time point of online cognitive assessments is being carried out approximately one year after the baseline assessments.

**Table 2:** PISA online cognitive assessment platforms.

Online platform	Task details	Approximate time to complete (min)	Reference
Cambridge Brain Sciences (CBS)	Twelve subtests of memory, reasoning, concentration and planning	30	(Corbett et al., 2015)
Cogstate	Online card games to test processing speed, attention (choice reaction time paradigm), visual memory (pattern separation paradigm) and working memory (n-back paradigm)	30 (including 15 for practise session and 15 for baseline session)	(Mielke et al., 2015)
Emotion Recognition	Participants label emotional facial expressions presented as morphs gradually expressing one of the six basic emotions	15	(Kessels et al., 2014)

#### 2.2.3 Genetic risk prediction

Genome-wide genotyping of participants within our recruitment pool was previously performed using a range of genotyping arrays including Illumina chips designed using HapMap references (317 K, 370 K, 610 K, 660 K) and more recent Illumina arrays designed using the 1KGP reference (Core + Exome, PsychArray, OmniExpress) as previously detailed (Cuellar-Partida et al., 2015, Medland et al., 2009). All datasets have been combined with strict quality control procedures and imputed to the Haplotype Reference Consortium (HRC) Release 1 reference panel (McCarthy et al., 2016).

##### 2.2.3.1 *APOE* genotyping

The three main *APOE* alleles- ε2, ε3 and ε4 -differ at two residues (rs429358 and rs7412) and so consist of a two single-nucleotide polymorphism (SNP) haplotype. We have previously ascertained the reliability of imputed *APOE* genotypes within our in-house dataset on each different genome-wide genotyping array used, using replication genotyping of 3576 samples (Lupton et al., 2018). If directly genotyped on the array or imputed with a genotype hard call imputation threshold of ≥0.9, and concordance threshold of >99.3% with our previous replication genotyping then we used the array based *APOE* genotype data. Samples not meeting these requirements were directly genotyped at the *APOE* locus using TaqMan SNP genotyping assays on an ABI Prism 7900HT and analyzed using SDS software (Applied Biosystems). SNPs were determined by allelic discrimination assays based on fluorogenic 5′ nuclease activity.

##### 2.2.3.2 Polygenic risk score (PRS)

PRS-no *APOE* was constructed from genome-wide SNP array data by summing the number of risk alleles weighted by the effect size (log odds ratio) as previously described (Lupton et al., 2017). Risk alleles were identified from the largest AD GWAS meta-analysis available at the start of the study, performed by the IGAP consortium (Lambert et al., 2013) (see Appendix C for details of the IGAP discovery sample). SNPs within 500 kb either side of the *APOE* locus were excluded to ensure all *APOE* associated signal was removed. A large number of SNPs associated with AD risk were included in the PRS, including variants associated at a level below genome-wide significance. A threshold of *p* ≤ 0.5 was chosen for inclusion in the PRS, as this has previously been shown to have the greatest predictive value (Escott-Price et al., 2015). Linkage disequilibrium (LD)-based clumping was carried out, providing the most significantly associated SNP in each region of LD (pairwise r^2^ threshold of 0.2 and a physical distance threshold of 300 kb).

### 2.3 PISA onsite

#### 2.3.1 PISA onsite overview

A subset of the online participant sample is selected for recruitment into the onsite arm of the study (PISA onsite). These include eligible individuals (criteria listed below) who are designated at high (N = 250) or low risk (N = 50) of AD based entirely on genetic classification. Those in the low risk group are in the lowest quintile of the AD PRS-no *APOE* (*APOE* gene region excluded) and are non *APOE* ɛ4 carriers. Those in the high risk group are either *APOE* ɛ4 carriers (including both homozygotes and heterozygotes), or those in the highest quintile of the PRS-no *APOE*. These groups, at either end of the AD genetic risk spectrum, were chosen to optimise the power to detect differences in biology and/or cognitive phenotype according to future AD risk. A detailed description of the genetic classification is given in the genetics section below.

In addition to the genetically enriched sample, 100 early stage or preclinical AD patients are being recruited from public and private memory clinics in Australia as comparative cases. These participants are recruited to provide a cohort of early AD changes against which to benchmark findings from (high-low) risk contrasts in our healthy cohort.

The process of physical, cognitive and neurobiological ageing will be closely monitored through the use of comprehensive examinations which cover five domains of investigation: multimodal imaging (PET and MRI), genetics, neuropsychology (cognition), smart sensing (lifestyle), and physiology. Participants will be followed up in a 2–3 year time window following for all baseline modalities, except for the PET scan. In brief, participants recruited into PISA onsite undergo thorough comprehensive examinations that quantify the structural and functional integrity of the brain, as well as the presence of neuropathology. Longitudinal neuropsychological, physiological and lifestyle data are also acquired. A bioinformatics database platform has been developed to capture all data, permitting multivariate and machine learning analyses to establish brain-behaviour, gene-brain, and gene-behavioural relationships and their mediation by AD genetic risk.

#### 2.3.2 Genetically enriched cohort participant selection

Participants were approached for recruitment into PISA onsite once they had completed at least the core module on the online survey. If a participant lived within a feasible travelling distance (approximately 100 km) of our testing site at QIMR Berghofer in Brisbane, they were sent information via email or mail about the PISA onsite study. This approach has yielded a 36% response rate. Once a participant expressed interest, they were screened for metal safety and eligibility, and if they met these criteria they were invited to make an appointment. Inclusion and exclusion criteria are shown in Box 1.

**Table:** 

Box 1. Criteria for recruitment of Healthy research participants into PISA onsite
**Inclusion Criteria** • Recruited from existing GWAS studies at QIMR Berghofer according to their genetic risk profile (see main text). Participants previously provided a DNA sample for GWAS and gave consent to participate in other ethically approved studies. • Aged 40–70 years • Fluent in English • Able to provide informed consent **Exclusion Criteria** • Any significant neurological disorder other than Alzheimer's disease (including stroke, vascular dementia, Parkinson’s disease, Huntington’s disease, normal-pressure hydrocephalus, CNS tumour or infection, seizure disorder, multiple sclerosis, or history of significant head trauma followed by haematoma, persistent neurological deficits or known structural brain abnormalities). • Any history of neurosurgery • Any significant medical condition that may confound neuropsychological testing (including chronic renal failure, chronic hepatic disease, severe pulmonary disease). • Current alcohol or substance (except tobacco) abuse or a past history of alcohol or substance (except tobacco) dependence. • History of severe psychiatric illness or current psychiatric symptoms that may confound neuropsychological testing (including major depression with psychotic symptoms, bipolar disorder or schizophrenia). • Females who are pregnant or breastfeeding. • Presence of prostheses or other objects that post a risk for MRI) including pacemaker, aneurysm clips, artificial heart valves, cochlear implants, metal fragments or foreign objects in the eyes, skin or body).

#### 2.3.3 Clinical cohort participant selection

The clinical cohort is recruited via local clinicians (neurologists, geriatricians, psychiatrists and general physicians) who see patients with cognitive concerns. Recruitment sites include the following health facilities in Brisbane: The Royal Brisbane and Women’s Hospital, The Wesley Hospital, The Mater Hospital, and Neurosciences Queensland, a private practice. Referrals are made to the team of PISA clinicians who then assess the suitability of referred patients according to clinical, neuropsychological and imaging criteria. PISA aims to recruit patients in the early stages of Alzheimer’s disease or whose clinical phenotype (aMCI) is suggestive of and highly likely to predict later diagnosis of Alzheimer’s disease. To facilitate the discovery of relevant AD biomarkers, patients with significant comorbidities (including, but not limited to major psychiatric illness or cerebrovascular disease) are excluded. If the inclusion criteria (Box 2) are met, patients are discussed at a consensus meeting between PISA clinicians, before determining their suitability for enrolment. The consensus meeting involves a review of case notes, available clinician correspondence, CT, MR and metabolic imaging (including SPECT and FDG-PET but not amyloid PET), and neuropsychological test results, where available.

**Table:** 

Box 2. Criteria for recruitment of Clinical research participants into PISA onsite
**Inclusion Criteria** • Meet DSM-5 Criteria for Mild Neurocognitive Disorder or Major Neurocognitive Disorder of the Alzheimer’s Type or NIA-AA Criteria for Mild Cognitive Impairment or Dementia of the Alzheimer’s type. • Mini-mental state examination (MMSE) > 20 and a Clinical Dementia Rating (CDR) of 0.5 or 1.0. *Exceptions to these criteria were made for clinically-probable cases of logopenic primary progressive aphasia (wherein language deficits might result in a lower MMSE) or posterior cortical atrophy (PCA) (wherein amnestic deficits may not be prominent).* • Age 40–80 years • Fluent in English • Able to provide informed consent • Exclusion Criteria • Any significant neurological disorder other than Alzheimer's disease (including stroke, vascular dementia, Parkinson’s disease, Huntington’s disease, normal-pressure hydrocephalus, CNS tumour or infection, seizure disorder, multiple sclerosis, and history of significant head trauma followed by haematoma, persistent neurological deficits or known structural brain abnormalities. • Any history of neurosurgery • Any significant medical condition that may confound neuropsychological testing (including chronic renal failure, chronic hepatic disease, severe pulmonary disease). • Current alcohol or substance (except tobacco) abuse or a past history of alcohol or substance (except tobacco) dependence. • History of severe psychiatric illness or current psychiatric symptoms that may confound neuropsychological testing (including major depression with psychotic symptoms, bipolar disorder or schizophrenia). • Females who are pregnant or breastfeeding. • Presence of prostheses or other objects that post a risk for MRI)including pacemaker, aneurysm clips, artificial heart valves, cochlear implants, metal fragments or foreign objects in the eyes, skin or body).

Following the first round of onsite PISA detailed neuropsychology and imaging (multi-dimensional MRI & amyloid PET) (described below), a second clinical consensus meeting is held to establish the blinded clinical diagnosis based on current criteria for MCI and AD (Albert et al., 2011, McKhann et al., 2011). Importantly, this occurs prior to divulging amyloid PET, which “unblinds” the clinicians to the presence or absence of amyloid deposition and may otherwise bias appraisal towards or away from a presumptive clinical diagnosis of AD.

#### 2.3.4 Onsite assessment overview

All participants, both genetically enriched and clinical cohorts, attend their first visit at QIMR Berghofer in Herston, QLD. After an overnight fast, the participant arrives at an allocated time and discusses the study in detail with a member of the research team before providing informed consent for all aspects of the study. A blood sample is donated and physical measures are collected, including height, weight, blood pressure and pulse (seated and standing), hip and waist measurements, and fat mass and percentage. Participants are provided with breakfast and are then walked a short distance to the Herston Imaging Research Facility (HIRF) where they participate in the MRI protocol and neuropsychological testing. The timing and order of MRI and Neuropsychological assessments is organised around imaging slots and neuropsychologist availability, to maximise the number of participants that can be assessed each day. All participants are invited to return two to three months later for the MRI amyloid PET scan. There is a low drop-out rate at this stage of the study, with 91% of participants returning for the amyloid PET scan. When participants attend their second visit, they receive a sleep sensing device to measure resting heart rate and sleep quality over five months.

#### 2.3.5 Multimodal imaging

A summary of the MRI parameters is shown in Table 3.

**MRI (PRISMA)**

**Table 3:** Summary of PISA MRI sequences. *Scans acquired on the PET/MRI.

Sequence Name	Acquisition Time(min:sec)	Matrix size (mm^3^)	Voxel size (mm^3^)	TE (msec)	TR (msec)	FA (degree)	TI (msec)
3D MP2RAGE	9:02	256 × 240 × 192	1 × 1 × 1	2.98	5000	4/5	701/2500
3D FLAIR	7:07	256 × 256 × 192	1 × 1 × 1	388	5000	4	1800
3D 9-echo-GRE	8:43	224 × 182 × 144	1 × 1 × 1	5.84 4.79 44.16	50	15	–
2D Diffusion	10:00	244 × 244 × 148	2 × 2 × 2	84	4700	90	–
2D Bold-Task fMRI	13:38	206 × 206 × 144	2.4 × 2.4 × 2.4	33	820	53	–
3D Dixon	0:26	320x220x144	1.2 × 1.2 × 3.0	1.29 2.52	3.97	9	–
3D MPRAGE*	5:03	256 × 240 × 192	1 × 1 × 1	2.26	2300	8	900
rs-fMRI *	10:05 (20:31)	220x220x42	3.1x3.1x3.0	30	2680	90	–
PC-ASL*	9:58	220x220x27	3.4x3.4x4.0	13	5100	90	–

Imaging data are acquired on a 3 T Siemens Prisma System (Siemens Healthineers, Erlangen, Germany) with the body coil for signal transmission and a 64-channel head coil and 18-channel body coil for signal reception (software version VE11). The following sequences are acquired:

T1-weighted structural brain images are acquired with the MP2RAGE sequence (TE/TR = 2.96 *ms*/5 *s*, TI2/TI2 = 0.701 s/2.5 s, FA1/FA2 = 4°/5°, 1 mm isotropic resolution, acquisition matrix 256 × 240 × 192, BW = 240 Hz/Px, 3xGRAPPA acceleration) (Marques et al., 2010). MP2RAGE imaging is used to increase the consistency of the longitudinal and multi-centric data compared to that provided by the MPRAGE sequence (Okubo et al., 2016). In addition, the MP2RAGE sequence provides T1 relaxation time measurements of brain tissues that are potential biomarkers of ageing and neurodegeneration (Tang et al., 2018).
T2-weighted fluid attenuation inversion recovery (FLAIR) images are included to assess white matter hyperintensity (WMH) burden, and parameters are as follows: TE/TR = 388/5000 *ms*, FA = 120°, acquisition matrix = 256 × 256 × 192, 1.0 *mm* voxel isotropic.
Multi-shell diffusion-weighted images (DWI) are acquired using parameters: TE/TR = 84/4700 *ms*, FOV 244 *mm*, acquisition matrix 122 × 122, 74 slices, slice thickness 2.0 *mm*, FA = 90°. The acquisition includes 12 non-diffusion weighted images (b = 0 *s/mm^2^*) as well as 20 (b = 1000 *s/mm^2^*), 32 (b = 2000 *s/mm^2^*), and 60 (b = 3000 *s/mm^2^*) unique directions. These are split across four blocks with alternating AP/PA phase encoding directions for the purpose of post-acquisition distortion correction.
A 3D gradient-recall echo (GRE) sequence are acquired with TE1/ΔTE/TE9/TR = 5.84/4.79/44.16/50 *ms*, FA = 15°, acquisition matrix 182 × 224 × 144, GRAPPA acceleration factor = 3, BW = 310 Hz/Px and flow compensation only to the first echo. The GRE sequence was acquired with true-axial orientation. The phase data from individual channels were combined properly using a reference image acquired by the body coil.
Task-based functional MRI data are acquired while participants view news clips. In brief, participants view the second half of 18 short news clips, having viewed the first half of 9 of these clips ten minutes earlier. These gradient echo data are acquired using the Siemens multi-slice acquisition with the following parameters: TR/TE = 820 ms/33.0 ms, flip angle = 53 deg, slices = 72, voxel size 2.4 × 2.4 × 2.4 mm, SMS factor = 6, acquisition direction PA plus two spin echo volumes with inverse PED (AP&PA) acquired for distortion correction. Brain regions activations are derived from the contrast of continuous (previous) viewing versus naïve (new) viewing. Further details of the task are provided in Appendix C.1.
In addition to the brain imaging, a two-point DIXON sequence is included to image the abdominal region between vertebral bodies T11 and L5 for assessment of visceral obesity. The acquisition is divided into two overlapping image slabs with the following parameters: TE1/TE2/TR = 1.29/2.52/3.97 *ms*, FA = 9°, acquisition matrix 320 × 220 × 144, BW = 1040 Hz/Px, voxel size 1.2 ×  × 1.2 *mm* and 3.0 *mm* slice thickness.
**Pet/MRI**

The positron emission tomography (PET) scans are performed on a Biograph mMR hybrid scanner (Siemens Healthineers, Erlangen, Germany) with Fluorine-18 florbetaben ([^18^F]FBB), a diagnostic radiotracer which possesses a highly selective binding for β-amyloid in neural tissue (Fodero-Tavoletti et al., 2012, Rowe et al., 2008).

With participants seated in a quiet room, 300 +/−10% MBq [18F]-Florbetaben is injected via an intravenous line inserted into a vein in the participant’s arm or hand. A 20-minute scan was acquired starting at 90 min post injection of [18F]-Florbetaben. The total PET/MR session takes 30 min to complete. PET counts are acquired using the Siemens mMR avalanche photodiodes (APDs).

For the concurrent MR, an MR UTE scan is first acquired for attenuation correction. Additionally, a T1-weighted structural image is acquired with the MPRAGE sequence (TE/TR = 2.26 *ms*/2.3 *s*, T1 = 0.9 *s*, FA = 8°, 1 mm isotropic resolution, matrix 256 × 240 × 192, BW = 200 Hz/Px, 2x GRAPPA acceleration) (Mugler and Brookeman, 1990). Two functional MRI sequences are then acquired in parallel with PET acquisition. From a subset of subjects, either a 20 min or 10 min resting state BOLD scan is acquired with a 2D echo planar sequence (TE/TR = 30 ms/2680 ms, FA = 90°, 3.1 × 3.1 × 3 mm resolution, matrix 220 × 220 × 42, BW = 2240 Hz/Px, 223 temporal images). From all other subjects, a 10 min rs-fMRI is acquired, followed by a 10 min resting state arterial spin labelling scan using a pseudo continuous arterial spin labelling with a 2D echo planar readout (TE/TR = 13 ms/5100 ms, post labelling delay = 1300 ms, labelling duration 1500 ms, FA = 90°, 3.4 × 3.4 × 4 mm resolution, matrix 220 × 220 × 27, 6/8 partial fourier, BW = 2298 Hz/Px, 117 temporal images). These sequences allow for motion correction of the PET images and also yield resting state BOLD and CBF images for independent analyses.

All imaging data are pre-processed using a common quality-controlled pipeline which integrates standard and customised steps (see Appendices sections C.2 and C.3 for further details)

**Imaging data storage**

DICOM images from the PRISMA and PET/MRI are collated and checked with in-house software (milxStager) for consistency with the predefined imaging protocol. DICOM data that passes consistency checks is then anonymised, converted to NIFTI and uploaded to an XNAT database (Marcus et al., 2007). RAW DICOMs are archived onto CSIRO servers. All subsequent processing is completed using data that is available on XNAT.

#### 2.3.6 Biological samples

**Fig. 2:**
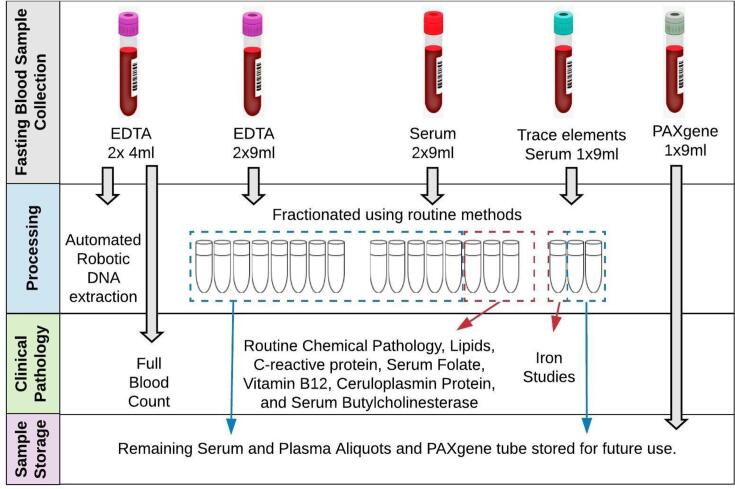
PISA Onsite blood sample collection and processing.

Participants in the PISA onsite cohort have blood samples collected to a maximum of 80 ml and utilised as shown in Fig. 2. Serum aliquots for biochemical testing are forwarded to a clinical pathology lab (Pathology Queensland, Royal Brisbane and Women’s Hospital). The full tests carried out are described in detail in Appendix D.

All samples are routinely *APOE* genotyped using the method described above, providing new data for the clinical cohort and confirming genotypes for the genetically enriched cohort. In addition clinical cohort samples are genotyped with a genome wide SNP array (Illumina Global Screening Array with Multi-disease drop-in, chip version GSAMD-24v1-0_20011747) in batches and imputed to the HRC r1.1 reference panel (McCarthy et al., 2016).

#### 2.3.7 Onsite neuropsychology

Cognitive and mood assessments for PISA Onsite participants are conducted by trained neuropsychologists. Participants complete a range of standardized tests selected to measure the main cognitive domains that typically decline in the typical amnestic form of AD and atypical non-amnestic presentations (language, visuospatial and executive dysfunction (McKhann et al., 2011)). We highlight that non-amnestic cognitive abilities are integral for memory test performance because executive functions such as attention and strategic processes are required to efficiently encode and retrieve memories and information to be recalled can be verbal or visual, implicating language and visuospatial cognitive skills. In addition, tests were chosen on the basis that they will enable comparison and harmonization with other large-scale and longitudinal studies; possess robust psychometric properties; and be sensitive to longitudinal change. A select number of tests were included as proxies for cognitive reserve (e.g. reading) or that are theoretically motivated by recent neuropsychological lesion studies which implicate left/right lateral prefrontal regions in specific executive functions (e.g., Fluency and Hayling Sentence Completion Tests; (Robinson et al., 2012, Robinson et al., 2015a)). Spontaneous speech samples are collected to measure language and propositional (idea) density; the number of ideas and connection or coherence between ideas (Barker et al., 2017). In the seminal Nun study, lower propositional density in younger adulthood was shown to predict poor cognitive performance in later life and, at autopsy, confirmed AD neuropathology (Snowdon et al., 1996). All tests are administered in person, then scored and transcribed (speech samples). Summary data is entered into spreadsheets with quality control checks.

The neuropsychological tests were selected based on their published psychometric properties (Table 4). Thus, we anticipate that each test will load primarily onto the domains they are listed under in Table 4. Of note, each domain comprises several tests, even if only one is explicitly identified. For example, visual perception is assessed by at least three measures (i.e., WASI-II Matrix Reasoning, cube analysis and TEA Telephone Search). Importantly, single neuropsychological test scores are required for the diagnosis of MCI and AD and all PISA participants (healthy and clinical) are checked against diagnostic criteria. Single test scores are also important and useful for theoretically derived analyses. Not all tests within each domain measure the same aspect of cognition. For example, while the Stroop task and Hayling Test are measures of verbal inhibition, the Hayling Test has both a semantic component and a measure of initiation, while the Stroop task is more visually based. Phonemic verbal fluency and semantic verbal fluency likewise are similar tasks although known to load differently onto the brain (Robinson et al., 2012). Comparing group performance on single tasks is a valid and useful approach to test theoretical questions of cognition and brain function while domain composite scores are useful and a robust way for predicting group fit (e.g. predicting amyloid status). From the list of single tests, researchers will be able to create composite scores within cognitive domains if that is appropriate for their particular analysis and research question.

**Table 4:** PISA onsite Neuropsychology assessment.

Domain	Tests
General Intelligence	Wechsler Abbreviated Scale of Intelligence –2^nd^Edition (WASI-II). 2 sub-tests: Similarities, Matrix Reasoning) (Wechsler, 2011)
Memory	Episodic Verbal - Rey Auditory Verbal Learning Test (Ivnik et al., 1990)
	Visual - Topographical Recognition Memory Test (Warrington, 1996)
	Working Memory: Wechsler Adult Intelligence Scale – Fourth Edition (WAIS-IV; Digit Span F/B) (Wechsler, 2003)
Language	Naming - Graded Naming Test (Warrington, 1997)
	Spontaneous speech - complex scene description – Beach Scene (Robinson et al., 2015b)
Literacy and Numeracy	Oral Graded-Difficulty Spelling Test (Baxter and Warrington, 1994)
	Oral-Graded-Difficulty Arithmetic Test (Jackson and Warrington, 1986)
	National Adult Reading Test (NART) (Nelson and Willison, 1991)
Executive Functions	Word fluency- FAS; Animals (Tombaugh et al., 1999)
	Stroop Test (Victoria version) (Troyer et al., 2006)
	Hayling Sentence Completion Test (Burgess and Shallice, 1997)
Psychomotor Speed	Symbol Digit Modality Test (Smith, 1973)
Visual Perception	Cube Analysis (VOSP) (Warrington and James, 1991)
Attention	TEA: Telephone Search; Dual Task (Robertson et al., 1994)
Social Cognition and Emotion Recognition	Mini-SEA emotion evaluation (Bertoux et al., 2014)
	TASIT-S part B Sarcasm (McDonald et al., 2017)
Current Mood Symptoms	Hospital Anxiety and Depression Scale (HADS) (Snaith and Zigmond, 1994)

All participants are asked screening questions about everyday function in activities and changes to functional or cognitive capacity to corroborate that they are in the healthy or clinical cohort. The length of assessment is typically two hours for healthy participants and three hours for the clinical cohort.

#### 2.3.8 Smart sensing

Recent research demonstrates a bidirectional relationship between sleep and AD pathology, with supporting evidence showing that changes in sleep patterns occur from the preclinical stage of AD (Ning and Jorfi, 2019). The co-occurrence of sleep disturbances and amyloid-beta (Aβ) accumulation suggest the importance of monitoring sleep patterns in adults who are at high-risk of developing neuro-degenerative disorders later in life.

Polysomnography (PSG) is the gold standard in measuring sleep parameters. However, there are several limitations in using PSG for longitudinal sleep monitoring. It is expensive, intrusive, and may lead to disturbed sleep patterns, due to an unfamiliar laboratory environment. Pervasive or ubiquitous sensors are an alternative choice for measuring sleep on a long-term basis due to low cost, and minimal obtrusion in a participant’s usual environment.

PISA onsite participants are given a contact-free sleep monitoring device when they return on-site for their PET scan. The EMFIT QS (Quantified Sleep) (Emfit, Filand) (Ranta et al., 2019) sleep monitor consists of a thin ferroelectric sensor that is placed underneath a bed mattress to perform at-home monitoring of sleep patterns. During their visit, participants are provided with verbal and written instructions on how to set up and use the device. The EMFIT QS connects to the participants’ home Wi-Fi and regularly transmits data to the research team. The device collects continuous resting heart rate and respiration rate, sleep stage estimates, sleep duration, sleep latency, waking rate and number of bed exits throughout the night. The heart rate and respiration rate measured by EMFIT QS has been validated against PSG measurements (Ranta et al., 2019).

So far, 168 PISA participants have been offered an EMFIT QS device. Of these, 102 participants completed the study, 50 participants declined due to Wi-Fi availability and/or privacy concerns, and 16 participants are currently using the device. The participants are given a reply-paid postage bag with the device and instructed to return the device when 5 months of data collection has been completed. The 5 month time frame allows a balance between the collection of representative data and the use of available sensors for additional participants.

The aim of the smart sensing stream is to understand the changes in sleep pattern across the spectrum of AD, from the preclinical stage to late-onset AD, and to investigate the impact of altered sleep in disease progression in AD. To do so, we introduce a sleep disruption index as a quantitative measure for significant changes in sleep patterns based on the collected data from the EMFIT QS.

#### 2.3.9 Bioinformatics

PISA phenotypic data are collated into a central RedCAP database (Harris et al., 2009). This includes basic MRI-derived measurements (such as hippocampal volume, white matter lesion volume, and ventricular volume) from imaging pipelines which are uploaded into RedCAP either as batch uploads or automatically at the end of the processing pipeline. Any manual annotations relevant to imaging acquisition (e.g. subject was claustrophobic, scan was cancelled after 20 min) are also uploaded. Results of the blood tests are automatically uploaded from HL7v2 files received from the pathology lab, preventing the need for manual entry and reducing data entry errors. All Additional PISA onsite phenotypic data are batch uploaded into RedCAP.

Measurement data in RedCAP is periodically locked down and cross checked to ensure no errors have propagated during measurements and data entry. This is carried out both manually and via automated scripts which examine the distribution of values and mark any outliers to be cross-checked. Data is made available for download to PISA investigators and collaborators using a web-based portal.

## 3 Results

### 3.1 Results overview

This is a first overview of our current sample sizes and demographic data as of the 21st of August 2020 from participants recruited for PISA online and the baseline of PISA onsite. Recruitment for both is ongoing, and the follow-up time point for PISA onsite (two years after the baseline visit) is now underway.

### 3.2 Results PISA online

**Fig. 3:**
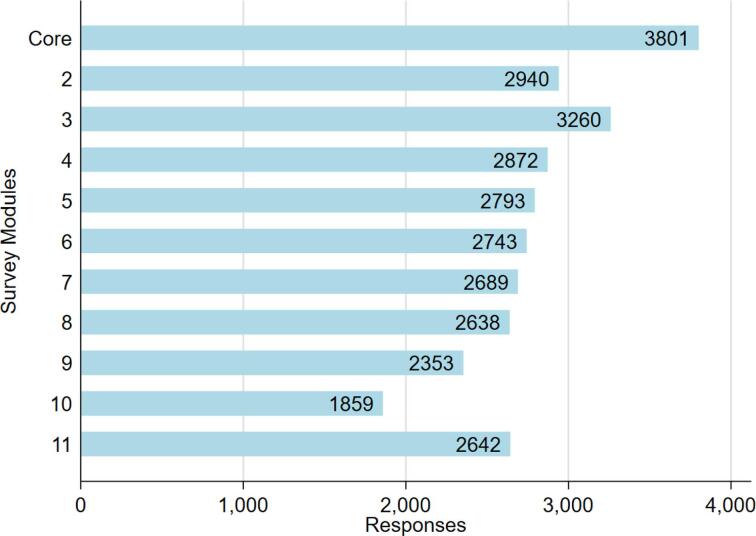
Participation in PISA online survey by module as of 21/08/20 (see Table 1 for module information and Appendix A for question details. Module 10 collects information on women’s health and therefore shows reduced participation because is completed by women only).

The online survey was launched on the 4th of July 2017. The current participation rate is 28%. To date, 3801 participants have taken part in the core survey with 65% of those going on to complete all additional modules (Fig. 3). The demographic information for participants who have completed the core module is given in Table 2. Ethnicity is predominantly Caucasian due to a prerequisite for Caucasian ancestry in the previous genetic studies which form the recruitment pool. Sample size will increase as the follow up of non-responders is ongoing, and includes provision of paper copies of the questionnaire if required.

Preliminary assessment of recruitment bias for PISA online shows significant associations of older age (β = 0.01, SE < 0.001, P < 0.001) and female sex (β = 0.01, SE < 0.001, P < 0.001) with participation in the study. No association was identified with the Alzheimer’s polygenic risk score (PRS) or *APOE ε4* carrier status.

Participants who have completed the online survey are also approached to complete online cognitive assessments. Currently 1956 participants have taken part in the CBS assessment (62% participation rate from 3176 who have been approached), 437 in Cogstate (26% of 1654 approached), and 927 completed the Emotion Recognition Task (56% of 1654 approached), with recruitment and follow-up ongoing. Low recruitment numbers for cogstate are partly due to a requirement for a computer (rather than tablet) and implementation problems with some operating systems on participants’ home computers.

### 3.3 Results PISA onsite baseline

To date 281 participants have completed baseline onsite assessments for PISA onsite. Of these 48 are clinical participants and 233 are genetically enriched (selected from those who have completed the online survey). Participant demographics are shown in Table 5.

**Table 5:** Participant demographics for PISA as of 21/08/20. For PISA online, data shown for all individuals who have completed the core module of the online survey.

	PISA Online N = 3801	PISA Onsite N = 281
Age*	Mean 60.3, SD 7.1, Min 41, Max 80,	Mean 60.9, SD 6.9, Min 43, Max 77
Sex	32.6% Male 67.4% Female	27.0% Male 73.0% Female
AD Diagnostic groups	N/A	29 AD 18 MCI 1 Other dementia 233 Genetically enriched
AD genetic risk Profiles	17.0% *APOE ɛ4* minor allele frequency 6.9% *APOE ɛ2* minor allele frequency	Genetically enriched only N = 233: N = 177 High genetic risk (*APOE ɛ*4 + ve AND/OR top quintile of PRS no-*APOE*), N = 56 Low genetic risk (*APOE ɛ*4 -ve AND lowest quintile of PRS no-*APOE*)
Ethnicity	98.1% Caucasian 0.2% Indigenous Australian/Torres Strait Islander 0.7%% Other/mixed ethnicity 0.9% Unknown	100% Caucasian
Years of Tertiary Education	Mean 13.2.0, SD 3.0, Max 28, Min 4	Mean 13.4, SD 3.0, Max 22, Min 8
Inclusion of Twin pairs	6.4% (122 pairs) dizygotic 9.6% (183 pairs) monozygotic	2 pairs dizygotic 9 pairs monozygotic
Current or past history of Neurological or psychiatric conditions	0.4% Alzheimer’s disease 0.1% Any other form of Dementia 0.3% Parkinson’s Disease 4.1% Memory complaints 2.1% Stroke/Transient Ischemic Attack/Mini-stroke 19.0% Anxiety disorder 19.8% Major depressive episode 1.0% Bipolar disorder 0.3% Schizophrenia	N/A
Lifestyle factors	18.4% History of hearing loss 16.7% Previous head injury (incl. loss consciousness) 30.9% History of hypertension 4.0% Current excessive alcohol consumption (>10 standard drinks** per week) 27.0% Obese (BMI ≥ 30) 42.5% Current or past smoker 5.5% History of type 2 diabetes 47.1% Perform vigorous physical activity (≥3 times per week)	N/A

**Age is age at MRI scan, a small subset of participants have an age greater than that specified in the recruitment criteria due to the delay between first contact and the onsite visit.

*Standard drinks according to the Australian standard, equivalent to 1.27 unit drink in the UK and 0.714 standard drinks in the USA.

Preliminary results for select baseline neuropsychology tests for the genetically enriched participants indicate a wide range of scores on the majority of tasks, with the mean of all tasks falling within or above the average range on all tasks based on the available published norms (Table 6). This provides confidence that our test battery is satisfactory and canvasses reasonable variability in cognitive function.

**Table 6:** PISA onsite baseline neuropsychology scores for the genetically enriched cohort.

Domain	Tests	M (SD)	Descriptor
General Intelligence	WASI-II IQ: Standard Score	104.26 (10.40)	*Average*
Memory	Rey Auditory Verbal Learning Test: Total Learning (z-score)	0.05 (1.05)	*Average*
	Topographical Recognition Memory: Test Total correct (Warrington, 1996)	26.08 (2.93)	*Average*/*High average*
	Digit Span Forward: Scaled Score	11.00	*Average*
Language	Graded Naming Test: Total correct	21.63 (3.44)	*Average*
	Spontaneous speech: Words per minute	130.17 (33.91)	*Within normal limits*
Literacy and Numeracy	Oral Graded-Difficulty Spelling Test: Total correct	23.70 (4.09)	*Average*
	Oral Graded-Difficulty Arithmetic Test Total correct	13.81 (5.04)	*Average*
	NART FSIQ Equivalent	109.30 (8.66)	*Average*/*High average*
Executive Functions	Word fluency FAS Total	42.37 (11.50)	*Average*
	Stroop Test Interference z-score	0.41 (0.72)	*Average*
	Hayling Sentence Completion Test: Overall Scaled Score	5.60 (1.19)	*Average*
Psychomotor Speed	Symbol Digit Modality Test Total correct	50.31 (8.63)	*Average*
Visual Perception	Cube Analysis: Total correct	9.71 (0.95)	*Within normal limits*
Attention	TEA: Telephone Search Scaled Score	11.50 (2.76)	*Average*
Social Cognition	Mini-SEA emotion evaluation Total correct	27.93 (3.03)	*Average*
Mood Symptoms	HADS Anxiety Score	5.47 (3.40)	*Within normal limits*

**Fig. 4:**
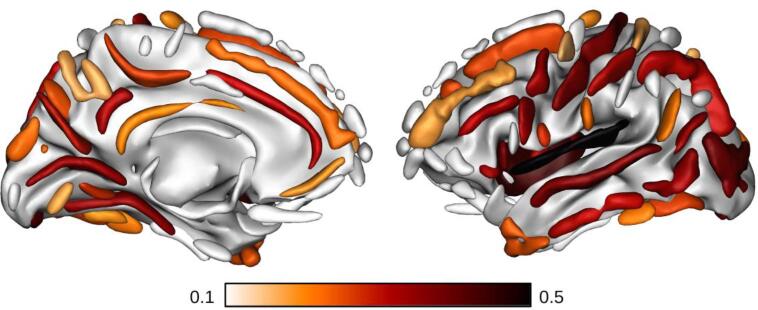
Pearson's correlation coefficient between age and sulcal width for the healthy cohort. Sulcal width was calculated using the Morphologist pipeline of the Brainvisa toolbox (Borne et al., 2020). Only significant correlation coefficients (two-tailed p-value<0.05 after Benjamini/Hochberg false discovery rate correction) are displayed. As expected, the retained sulci have a positive correlation coefficient.

As an illustrative analysis of the baseline MRI data, we regressed sulcal width against age (and age squared) for the 202 healthy participants (genetically enriched cohort) of which the T1w MPRAGE MRI scans are currently available (Fig. 4). In particular, the width of 64 individual sulci were extracted using the Morphologist pipeline of the Brainvisa toolbox (Borne et al., 2020). Correlations against age and age squared were derived across the sample and corrected for multiple comparisons using the Benjamini-Hochberg false discovery rate correction. Consistent with prior work (Good et al., 2001), the effect of age on cortical anatomy is widespread, although heterogenous. 43 of the sulci (out of 64) are significantly correlated with age (vs. 44 for age squared). Increasing width with age was strongest in the insula, lateral fissure, precentral sulcus, calcarine fissure and superior temporal sulcus. The regression against the age squared term has a positive coefficient and thus suggests an accelerating rate of sulci expansion in this population.

The follow-up time point phenotyping is currently underway, with 104 participants currently approached, giving an 88% retainment rate thus far (92 participants have completed follow-up assessments, 2 have become ineligible and 10 have withdrawn).

## 4 Discussion

The primary outcome of the PISA study is to establish a longitudinal cohort of healthy midlife-elderly individuals at high genetic risk of dementia. This will be a unique international resource – only enabled by the recent breakthroughs in genetic analysis – and will yield new possibilities for basic and translational research. This protocol and cohort are of broad significance for other investigations – particularly clinical trials in dementia prevention.

PISA is not designed to test specific hypotheses regarding genetic risk, phenotype and brain, but rather to complement other discovery-oriented cohort studies such as the UK biobank: Focussing specifically on AD allows deeper phenotyping and imaging of AD-specific features. As such, the size of the cohort (currently N = 281, of which 233 are from the genetically enriched arm) was not predetermined by a specific power calculation. However, recent studies suggest PISA is well powered to detect core candidate effects. For example, slower rates of hippocampal atrophy and higher levels of CSF Aβ were recently detected in cognitively normal older *APOE* ε2 carriers (*N* = 27 *APOE* ε2/ε2 or ε2/ε3, versus *N* = 107 *APOE* ε3/ε3) (Chiang et al., 2010); Relative to Aβ+ ɛ4 noncarriers (*N* = 36), Aβ+ ɛ4 carriers (*N* = 48) showed significantly faster decline on memory tasks (Lim et al., 2015). An association between AD PRS and smaller left hippocampal volume, remained when the *APOE* gene was excluded (*N =* 272) (Foley et al., 2017). Similarly, AD-specific cortical thinning was correlated with the AD polygenic risk score, even after controlling for *APOE* genotype and cerebrospinal fluid (CSF) levels of β-amyloid (*N* = 104) (Sabuncu et al., 2012).We also aim to progressively increase the sample size of PISA through ongoing recruitment, and lengthen and increase longitudinal sampling, subject to funding.

The age range of the genetically enriched cohort spans from midlife to older adults, encompassing a range of individuals at different levels of risk for AD, with a considerable variance IQ and cognitive function. Recent work has shown that high levels of neocortical Aβ-amyloid occur at age 72, but earlier (at age 63) for *APOE ε4* carriers (Burnham et al., 2020), suggesting that our healthy cohort should also include a substantial proportion of individuals with Aβ-amyloid. Using advanced structural, functional, and molecular imaging technologies, this will enable characterizing the neurobiological features associated with high risk for dementia and, in particular, identify those changes associated with the initial onset of cognitive impairment in those high-risk participants as they begin the transition to AD. Together with genetic risk prediction, such knowledge has clear potential to develop prognostic markers for dementia development.

While we study the interplay between genetic and environmental factors for dementia, we also aim to identify those risk factors (e.g. lifestyle) that could be modified through intervention. Recent evidence points to several important modifiable risk factors for AD including education level, hypertension, hearing impairment, smoking, obesity, depression, physical inactivity, diabetes, low social contact, excessive alcohol consumption, traumatic brain injury, and air pollution (Livingston et al., 2020). The multimodal imaging, behavioural, neuropsychological and sleep data will be a unique resource to assist in understanding the complex and dynamic relationship between neurobiological antecedents of dementia at high risk and the subsequent progression to cognitive impairment. In addition, the acquisition and storage of DNA, RNA (PAX gene tube), serum and plasma will allow for future work including epigenetics, gene expression, and blood based biomarkers.

Limitations of the PISA study include that the genetically enriched cohort is selected from a database of previous research participants who originally consented to be part of a prior research study, allowed re-contact and then consented to take part in PISA. This multi-stage recruitment process will likely inflate any selection bias related to willingness to participate in research studies. In addition, characteristics of the original studies’ participant selection will be reflected in PISA participants including geographical locations, the inclusion of twins and relatives of twins, the large proportion of female participants (due to increased rates of females volunteering for research over males, over two recruitment occasions) and the predominance of Australians of Caucasian ethnicity. For the PISA onsite genetically enriched cohort individuals who are at both high and low genetic risk of AD were purposely selected. Therefore, rather than a traditional case/control design where the controls are representative of the normal population, this method reflects a kind of ‘extreme sampling’ in terms of genetic risk. For replication purposes, samples can be selectively drawn from other large-scale cohorts, for example UK biobank (Sudlow et al., 2015), ADNI (Mueller et al., 2005) and AIBL (Ellis et al., 2009).

In summary, PISA is a genetically enriched study of healthy and mid-life adults at high risk of future dementia, funded by the National Health and Medical Research Council. Current funding permits the full baseline acquisition and follow-up at 2 years, on a par with other studies including AIBL (AD case control & MCI, with an 18mnth follow-up (Ellis et al., 2009)), The Older Australian Twin Study (OATS, elderly population based with a 2 year follow-up (Sachdev et al., 2009)) and the Sydney Memory and Aging Study (MAS, elderly population based, also with a 2 year follow-up (Sachdev et al., 2010)). We expect this 2 year time difference sufficient to detect the strongest within-subject effect of polygene risk on cognitive and structural brain change. Charting nonlinear time-related difference and detecting more subtle effects will indeed require cohort expansion and further follow-up. Achieving our future aim to continue over subsequent waves of data collection and expand on baseline sample size will require additional funding and collaboration.

## 5 Data availability

Following completion of each wave (baseline, follow-up) and appropriate quality control, de-identified PISA data will be made available to other research groups upon request. Due to privacy, confidentiality and constraints imposed by the local Human Research Ethics Committee, a “Data Sharing Agreement” will be required before data will be released. Due to ethics constraints, data will be shared on a project-specific basis. Depending on the nature of the data requested, evidence of local ethics approval may be required.

## CRediT authorship contribution statement

**Michelle K. Lupton:** Conceptualization, Methodology, Formal analysis, Investigation, Writing - review & editing, Supervision. **Gail A. Robinson:** Conceptualization, Methodology, Formal analysis, Investigation, Writing - review & editing, Funding acquisition. **Robert J. Adam:** Resources, Writing - review & editing. **Stephen Rose:** Conceptualization, Writing - review & editing, Funding acquisition. **Gerard J. Byrne:** Resources, Writing - review & editing, Funding acquisition. **Olivier Salvado:** Conceptualization, Writing - review & editing, Funding acquisition, Funding acquisition. **Nancy A. Pachana:** Conceptualization, Writing - review & editing, Funding acquisition. **Osvaldo P. Almeida:** Conceptualization, Writing - review & editing, Funding acquisition. **Kerrie McAloney:** Investigation, Data curation, Writing - review & editing, Supervision, Project administration. **Scott D Gordon:** Data curation, Writing - review & editing. **Parnesh Raniga:** Data curation, Writing - review & editing. **Amir Fazlollahi:** Formal analysis, Writing - review & editing. **Ying Xia:** Formal analysis, Writing - review & editing. **Amelia Ceslis:** Formal analysis, Investigation, Data curation, Writing - review & editing. **Saurabh Sonkusare:** Investigation, Writing - review & editing. **Qing Zhang:** Formal analysis, Writing - review & editing. **Mahnoosh Kholghi:** Formal analysis, Investigation, Writing - review & editing. **Mohan Karunanithi:** Formal analysis, Writing - review & editing. **Philip E Mosley:** Formal analysis, Resources, Writing - review & editing. **Jinglei Lv:** Formal analysis, Writing - review & editing. **Léonie Borne:** Formal analysis, Writing - review & editing. **Jessica Adsett:** Investigation, Data curation, Writing - review & editing. **Natalie Garden:** Investigation, Data curation, Writing - review & editing. **Jurgen Fripp:** Methodology, Writing - review & editing, Supervision, Funding acquisition. **Nicholas G. Martin:** Conceptualization, Methodology, Resources, Writing - review & editing, Supervision, Funding acquisition. **Christine C Guo:** Conceptualization, Methodology, Formal analysis, Investigation, Writing - review & editing, Supervision, Funding acquisition. **Michael Breakspear:** Conceptualization, Methodology, Formal analysis, Writing - review & editing, Supervision, Funding acquisition.

## Declaration of Competing Interest

The authors declare that they have no known competing financial interests or personal relationships that could have appeared to influence the work reported in this paper.
