# Supplementary material for: A prospective cohort study of prodromal Alzheimer’s disease: Prospective Imaging Study of Ageing: Genes, Brain and Behaviour (PISA)
Source: Neuroimage Clin. 2020 Dec 8;29:102527. doi: 10.1016/j.nicl.2020.102527 (PMC7750170; doi:10.1016/j.nicl.2020.102527)
Supplement: Supplementary data 1 [file mmc1.pdf]

Appendices

A. PISA Online Survey instruments

A.1 Core Module

Questionnaire Name	Reference	Items	Inclusions
Key demographics	QIMRB in-house	32	Key demographic information such as DOB, postcode, education, gender, etc.
Extended family	QIMRB in-house	120	Parents' age or death details, number of siblings and children
Biological Family Medical History	QIMRB in-house	32	Medical history of self and biological family members
Memory and Health	QIMRB in-house	11	Self-rated memory, physical and mental health, and sleep
Substance use	QIMRB in-house	18	Covers alcohol, drugs and smoking history
Patient Health Questionnaire (PHQ-9)	Kroenke et al., 2001	10	Screening, diagnosing, monitoring and measuring the severity of depression
Generalised Anxiety Disorder (GAD-7)	Spitzer et al., 2006	8	Screening tool and severity measure for generalised anxiety disorder
Medications	QIMRB in-house	3	Common medications for AD or dementia, and psychiatric disorders
Active Australia Survey (modified)	Australian Institute of Health and Welfare, 2003	13	Measures participation in physical activity and knowledge of current public health messages about the health benefits of physical activity
Personal medical history	QIMRB in-house	23	Screening questions on psychiatric illness, brain injury, heart surgery etc.

A.2 Memory & Cognition

Questionnaire Name	Reference	Items	Inclusions
Early cognitive history	QIMRB in-house	8	Learning disorders, developmental delays, significant trauma or repeating a grade during early life
Stutter	QIMRB in-house	1	Self-rating of stutter
E-Cog	Farias et al., 2008	39	Assesses the functional abilities of older adults across a wide range of ability spanning normal ageing through to dementia
HBA Functional Assessment Tool	Mowszowski et al. (2017)	45	Assessment of cognition and functional status

A.3 Medical History

Questionnaire Name	Reference	Items	Inclusions
Psychological history	QIMRB in-house	61	Any diagnosed psychiatric condition and specific treatment history
Medical conditions	QIMRB in-house	85	Heart conditions and other specific medical conditions
Whiteley-7 Scale	Fink et al., 1999	7	Screening instrument for somatization illness
Chronic pain	QIMRB in-house	2	Measures experience and intensity of chronic pain
Back pain	QIMRB in-house	3	Experience and intensity of lower back pain
Asthma and eczema	QIMRB in-house	17	Past history or current diagnosis of asthma, wheezing, coughing, hayfever, pneumonia
Allergies	QIMRB in-house	19	Allergies and subsequent reactions to them
Migraine	QIMRB in-house	29	History of migraines, relation to specific triggers
Falls Risk Questionnaire	Rubenstein et al., 2011	12	Assesses a person's risk of falling
Monthly Falls Questionnaire	Lord et al., 2001	19	Looks at falls over the past month and where they occurred, how they happened and the outcomes

A.4 Personal Wellbeing

Questionnaire Name	Reference	Items	Inclusions
Somatic and Psychological Health Report (SPHERE-12)	Hickie et al., 200)	12	Psychological and physical health
Kessler Psychological Distress Scale (K-10)	Kessler et al., 2002	10	Symptoms of anxiety and depression
Satisfaction with Life Scale (SWLS)	Diener et al., 1985	5	Global cognitive judgments of satisfaction with one's life
Personal Wellbeing Index (PWI)	International Wellbeing Group, 2013	9	Subjective dimension of quality of life known as 'subjective wellbeing'
Depression screen	QIMRB in-house	19	Screening questions for diagnosed depression
Anxiety screen	QIMRB in-house	9	Screening questions for diagnosed anxiety
Psychosis screen	QIMRB in-house	16	Screening questions for diagnosed psychosis
PTSD screen	QIMRB in-house	63	Screening questions for diagnosed PTSD
Sexuality questions	QIMRB in-house	2	Sexuality preferences and age of first sexual experience

A.5 Lifestyle

Questionnaire Name	Reference	Items	Inclusions
Victoria Longitudinal Study Activities Questionnaire	Hultsch et al., 1999	70	Measures participation in a range of activities over the last 2 years
Substance use	QIMRB in-house	100	Consumption of alcohol, tobacco or illicit drugs over lifetime
Substance abuse criteria	QIMRB in-house	272	Screening for substance abuse past or current

A.6 Personality

Questionnaire Name	Reference	Items	Inclusions
Neuroticism Extraversion Openness Five Factor Inventory-3 (NEO-FFI-3)	Costa and McCrae, 199)	60	Personality test based on 5-factor model of neuroticism, extraversion, openness, agreeableness and conscientiousness
International Personality Disorder Examination (IPDE)	Loranger et al., 1997	59	Personality disorder screen for following personality types: Paranoid, schizoid, dissocial, impulsive, borderline, histrionic, anankastic, anxious, dependent
Buss Perry Aggression Questionnaire	Buss and Perry, 1992	29	Aggression and violent behaviour, aggressive thoughts

A.7 Life events

Questionnaire Name	Reference	Items	Inclusions
Being a twin	QIMRB in-house	62	A range of birth, childhood and adulthood experiences relating to twins
Ethnicity and Ancestry	QIMRB in-house	5	Ethnicity and ancestry of parents and grandparents
Recent life changes stress test	Miller and Rahe, 1997	81	Information on life changes and the impact of stressful life events on health
Early life/childhood	QIMRB in-house	13	Financial status and educational effects
Mindsets Test	QIMRB in-house	8	Perceptions and beliefs about intelligence and learning
Present Life Attitudes	QIMRB in-house	7	Attitudes and engagement towards life and ageing
Lifetime pollution exposure	QIMRB in-house	106	Address details from last 30 years to assess exposure to pollution

A.8 Feelings and Emotions

Questionnaire Name	Reference	Items	Inclusions
Positive and Negative Affect Schedule (PANAS)	Watson et al., 1988	20	Rating of feelings and emotions experienced currently or over the last week
Perceived Stress Scale (PSS)	Cohen et al., 1983	10	Feelings, thoughts and experiences of stress over the past month
Geriatric Anxiety Inventory (GAI)	Pachana et al., 2007	20	Assessment of anxiety in older adults
Geriatric Depression Scale (GDS)	Sheikh and Yesavage, 1986	15	Assessment of depressive symptoms in older adults
Adult ADHD Self-Report Scale (ASRS-v1.1)	Kessler et al., 2005	18	Checklist of ADHD symptoms based on diagnostic criteria within the DSM-IV
Personality Assessment Inventory: Borderline Features (PAI-BOR)	Morey, 1991	24	Covers attributes indicative of a borderline personality
Social Responsiveness Scale (SRS)	Constantino and Gruber, 2012	11	Presence and severity of social impairment within the autism spectrum. Subset of questions adapted from full questionnaire of 65 items.
Barkley Adult ADHD Rating Scale (BAARS-IV)	Barkley, 2011	12	Checklist of common adult ADHD symptoms, subset of questions adapted from Barkley Adult ADHD Rating Scale

A.9 Physical Health

Questionnaire Name	Reference	Items	Inclusions
Physical characteristics	QIMRB in-house	20	
Short Form Health Survey (SF-36)	Ware et al., 1993	36	Profile of functional health and wellbeing scores, psychometrically-based physical and mental health summary measures and a preference-based health utility index
Pittsburgh Sleep Quality Index (PSQI)	Buysse et al., 1989	24	Quality, quantity and patterns of sleep in adults
Napping	QIMRB in-house	1	Length of regular naps if applicable
Berlin Questionnaire for Sleep Apnoea	Netzer et al., 1999	10	Presence or absence of Obstructive Sleep Apnoea
Nutrition History Questionnaire	Hark and Deen, 1999	32	Overall sense of patient's daily eating habits
Eating Disorder Screen	QIMRB in-house	3	Screen for symptoms of eating disorders such as anorexia nervosa or binge-eating disorder

A.10 Women’s Health

Questionnaire Name	Reference	Items	Inclusions
Menstruation	QIMRB in-house	1	Age of first menstruation
Pregnancy	QIMRB in-house	63	Number of pregnancies, and symptoms or issues throughout each
Births	QIMRB in-house	31	Number of births and demographic information relating to each instance
Morning sickness	QIMRB in-house	5	Presence and severity of morning sickness during pregnancy
Breastfeeding	QIMRB in-house	6	Length of time spent breastfeeding for each birth
Depression	QIMRB in-house	24	Presence of depression during or following pregnancy
Menopause	QIMRB in-house	4	Age of onset of menopause and relation to HRT or surgery
Endometriosis	QIMRB in-house	7	Endometriosis diagnosis status

A.11 Pain

Questionnaire Name	Reference	Items	Inclusions
General pain questions	QIMRB in-house	6	Experiences of major and current pain
Graded Chronic Pain Scale	Von Korff et al., 1992	6	Chronic pain in the last 6 months
Vitamins and supplements	QIMRB in-house	60	Vitamins and supplements taken regularly in the last 12 months
Osteoarthritis Brief Questionnaire	QIMRB in-house	8	Experiences of osteoarthritis

A.12 Online Survey instrument References

Australian Institute of Health and Welfare, 2003. The Active Australia Survey: a guide and manual for implementation, analysis and reporting. Australian Institute of Health and Welfare, Canberra.

Barkley, R., 2011. Barkley Adult ADHD Rating Scale-IV (BAARS-IV). The Guildford Press, New York.

Buss, A., Perry, M., 1992. The Aggression Questionnaire. Journal of Personality and Social Psychology 63(3), 452-459.

Buysse, D., Reynolds, C., Monk, T., Berman, S., Kupfer, D., 1989. The Pittsburgh Sleep Quality Index: a new instrument for psychiatric practice and research. Psychiatry Research 28(2), 193-213.

Cohen, S., Kamarck, T., Mermelstein, R., 1983. A global measure of perceived stress. Journal and Health and Social Behavior 24(4), 385-396.
Constantino, J., Gruber, C., 2012. Social Responsiveness Scale, 2nd ed. Western Psychological Services, Los Angeles, CA.

Costa, P., McCrae, R., 1992. Revised NEO personality inventory (NEO-PI-R) and NEO five-factor inventory (NEO-FFI): Professional manual. Psychological Assessment Resources, Inc. , Odessa, FL.

Diener, E., Emmons, R.A., Larsen, R.J., Griffin, S., 1985. The Satisfaction With Life Scale. Journal of Personality Assessment 49(1), 71-75.

Farias, S.T., Mungas, D., Reed, B.R., Cahn-Weiner, D., Jagust, W., Baynes, K., DeCarli, C., 2008. The Measurement of Everyday Cognition (ECog): Scale Development and Psychometric Properties. Neuropsychology 22(4), 531-544.

Fink, P., Ewald, H., Jensen, J., Sorensen, L., Engberg, M., Holm, M., Munk-Jorgensen, P., 1999. Screening for somatization and hypochondriasis in primary care and neurological in-patients: a seven-item scale for hypochondriasis and somatization. Journal of Psychosomatic Research 46(3), 261-273.

Hark, L., Deen, D., 1999. Taking a Nutrition History: A Practical Approach for Family Physicians. American Family Physician 59(6), 1521-1528.

Hickie, I.B., Davenport, T.A., Hadzi-Pavlovik, D., Koschera, A., Naismith, S.L., Scott, E.M., Wilhelm, K.A., 2001. Development of a simple screening tool for common mental disorders in general practice. Medical Journal of Australia 175(S1), 175-S110-177.

Hultsch, D., Hertzog, D., Small, B., Dixon, R., Backman, L., Nilsson, L., 1999. Use it or lose it: Engaged lifestyle as a buffer of cognitive decline in aging? Psychology and Aging 14(2), 245-263.

International Wellbeing Group, 2013. Personal Wellbeing Index: 5th Edition. Australian Centre on Quality of Life, Deakin University, Melbourne.

Kessler, R., Adler, L., Ames, M., Demler, O., Faraone, S., Hiripi, E., Howes, M., Jin, R., Secnik, K., Spencer, T., Ustun, T., Walters, E., 2005. The World Health Organization Adult ADHD Self-Report Scale (ASRS): a short screening scale for use in the general population. Psychological Medicine 35(2), 245-256.

Kessler, R., Andrews, G., Colpe, L., Hiripi, E., Mroczek, D., Normand, S., Walters, E., Zaslavksy, A., 2002. Short screening scales to monitor population prevalences and trends in non-specific psychological distress. Psychological Medicine 32(6), 959-976.

Kroenke, K., Spitzer, R.L., Williams, J.B., 2001. The PHQ-9: Validity of a Brief Depression Severity Measure. Journal of General Internal Medicine 16(9), 606-613.

Lambert, J.C., Ibrahim-Verbaas, C.A., Harold, D., Naj, A.C., Sims, R., Bellenguez, C., Jun, G., Destefano, A.L., Bis, J.C., Beecham, G.W., Grenier-Boley, B., Russo, G., Thornton-Wells, T.A., Jones, N., Smith, A.V., Chouraki, V., Thomas, C., Ikram, M.A., Zelenika, D., Vardarajan, B.N.,

Kamatani, Y., Lin, C.F., Gerrish, A., Schmidt, H., Kunkle, B., Dunstan, M.L., Ruiz, A., Bihoreau, M.T., Choi, S.H., Reitz, C., Pasquier, F., Hollingworth, P., Ramirez, A., Hanon, O., Fitzpatrick, A.L., Buxbaum, J.D., Campion, D., Crane, P.K., Baldwin, C., Becker, T., Gudnason, V., Cruchaga, C., Craig, D., Amin, N., Berr, C., Lopez, O.L., De Jager, P.L., Deramecourt, V., Johnston, J.A., Evans, D., Lovestone, S., Letenneur, L., Moron, F.J., Rubinsztein, D.C., Eiriksdottir, G., Sleegers, K., Goate, A.M., Fievet, N., Huentelman, M.J., Gill, M., Brown, K., Kamboh, M.I., Keller, L., Barberger-Gateau, P., McGuinness, B., Larson, E.B., Green, R., Myers, A.J., Dufouil, C., Todd, S., Wallon, D., Love, S., Rogaeva, E., Gallacher, J., St George-Hyslop, P., Clarimon, J., Lleo, A., Bayer, A., Tsuang, D.W., Yu, L., Tsolaki, M., Bossu, P., Spalletta, G., Proitsi, P., Collinge, J., Sorbi, S., Sanchez-Garcia, F., Fox, N.C., Hardy, J., Naranjo, M.C., Bosco, P., Clarke, R., Brayne, C., Galimberti, D., Mancuso, M., Matthews, F., European Alzheimer's Disease, I., Genetic, Environmental Risk in Alzheimer's, D., Alzheimer's Disease Genetic, C., Cohorts for, H., Aging Research in Genomic, E., Moebus, S., Mecocci, P., Del Zompo, M., Maier, W., Hampel, H., Pilotto, A., Bullido, M., Panza, F., Caffarra, P., Nacmias, B., Gilbert, J.R., Mayhaus, M., Lannfelt, L., Hakonarson, H., Pichler, S., Carrasquillo, M.M., Ingelsson, M., Beekly, D., Alvarez, V., Zou, F., Valladares, O., Younkin, S.G., Coto, E., Hamilton-Nelson, K.L., Gu, W., Razquin, C., Pastor, P., Mateo, I., Owen, M.J., Faber, K.M., Jonsson, P.V., Combarros, O., O'Donovan, M.C., Cantwell, L.B., Soininen, H., Blacker, D., Mead, S., Mosley, T.H., Jr., Bennett, D.A., Harris, T.B., Fratiglioni, L., Holmes, C., de Bruijn, R.F., Passmore, P., Montine, T.J., Bettens, K., Rotter, J.I., Brice, A., Morgan, K., Foroud, T.M., Kukull, W.A., Hannequin, D., Powell, J.F., Nalls, M.A., Ritchie, K., Lunetta, K.L., Kauwe, J.S., Boerwinkle, E., Riemenschneider, M., Boada, M., Hiltunen, M., Martin, E.R., Schmidt, R., Rujescu, D., Wang, L.S., Dartigues, J.F., Mayeux, R., Tzourio, C., Hofman, A., Nothen, M.M., Graff, C., Psaty, B.M., Jones, L., Haines, J.L., Holmans, P.A., Lathrop, M., Pericak-Vance, M.A., Launer, L.J., Farrer, L.A., van Duijn, C.M., Van Broeckhoven, C., Moskvina, V., Seshadri, S., Williams, J., Schellenberg, G.D., Amouyel, P., 2013. Meta-analysis of 74,046 individuals identifies 11 new susceptibility loci for Alzheimer’s disease. Nat Genet 45(12), 1452-1458.

Loranger, A.W., Janca, A., Sartorius, N., World Health Organization, 1997. Assessment and diagnosis of personality disorders: the ICD-10 international personality disorder examination (IPDE). Cambridge University, Cambridge.

Lord, S.R., Sherrington, C., Menz, H.B., 2001. Falls in Older People: Risk factors and strategies for prevention. Cambridge University Press, Cambridge.
Miller, M., Rahe, R., 1997. Life changes scaling for the 1990s. Journal of Psychometric Research 43(3), 279-292.

Morey, L., 1991. Personality Assessment Inventory professional manual. Psychological Assessment Resources, Odessa, FL.

Mowszowski, L., LaMonica, H., Naismith, S., 2017. Detecting subtle functional decline in prodromal dementia, Australian Dementia Forum, NHMRC National Institute for Dementia Research. Melbourne, Australia.

Netzer, N., Stoohs, R., Netzer, C., Clark, K., Strohl, K., 1999. Using the Berlin Questionnaire to Identify Patients at Risk for the Sleep Apnea Syndrome. Annals of Internal Medicine 131, 485-491.

Pachana, N., Byrne, G., Siddle, H., Koloski, N., Harley, E., Arnold, E., 2007. Development and validation of the Geriatric Anxiety Inventory. International Psychogeriatrics 19(1), 103-114.

Rubenstein, L.Z., Vivrette, R., Harker, J.O., Stevens, J.A., Kramer, B.J., 2011. Validating an evidence-based, self-rated fall risk questionnaire (FRQ) for older adults. Journal of Safety Research 42(6), 493-499.

Sheikh, J., Yesavage, J., 1986. Geriatric Depression Scale (GDS): Recent evidence and development of a shorter version, in: Brink, T. (Ed.) Clinical Gerontology: A Guide to Assessment and Intervention. The Haworth Press, Inc., New York.

Spitzer, R.L., Kroenke, K., Williams, J.B., Lowe, B., 2006. A brief measure for assessing generalized anxiety disorder: the GAD-7. Archives of Internal Medicine 166(10), 1092-1097.

Von Korff, M., Ormel, J., Keefe, F., Dworkin, S., 1992. Grading the severity of chronic pain. Pain 50(2), 133-149.

Ware, J., Snow, K., Kosinski, M., Gandek, B., 1993. SF-36 Health Survey Manual and Interpretation Guide. New England Medical Centre, The Health Institute, Boston, MA.

Watson, D., Clark, L., Tellegan, A., 1988. Development and validation of brief measures of positive and negative affect: the PANAS scales. Journal of Personality and Social Psychology 54(6), 1063-1070.

B. IGAP consortium discovery sample

Polygenic risk scores (PRS) were calculated using results data from the International Genomics of Alzheimer's project (IGAP) as the discovery sample (Lambert et al., 2013). This is large two-stage study based upon genome-wide association studies (GWAS) on individuals of European ancestry. In stage 1, IGAP used genotyped and imputed data on 7,055,881 single nucleotide polymorphisms (SNPs) to meta-analyse four previously-published GWAS datasets consisting of 17,008 Alzheimer's disease cases and 37,154 controls (The European Alzheimer's disease Initiative – EADI the Alzheimer Disease Genetics Consortium – ADGC The Cohorts for Heart and Aging Research in Genomic Epidemiology consortium – CHARGE The Genetic and Environmental Risk in AD consortium – GERAD). In stage 2, 11,632 SNPs were genotyped and tested for association in an independent set of 8,572 Alzheimer's disease cases and 11,312 controls. Finally, a meta-analysis was performed combining results from stages 1 & 2.

C. MRI Processing Methods

C.1 Functional MRI task details

News clips were purchased from Australian Broadcasting Corporation (ABC), on different topics including animal, local, politics, sports, science and business. The length of news clips ranged from 31 to 59 s (45.06 ± 6.67). Each clip was segmented

at an existing scene cut into two halves of approximately similar length. Nine clips were selected for the continuing condition (clips of which both the first and second halves are shown) and nine for the naïve condition (clips of which only the second halves are shown), matched for topic, valence, gender of newscaster and clip length. While acquiring non-fMRI data, the first halves of clips in continuing condition were shown. After a 10 min delay, the second halves of all 18 news clips were shown to the participants. All news clips were interleaved by fixation periods of 10–12 s. The order of clips within each session (initial and continuing) was randomised for each participant. The contrast of continuing versus naïve clips elicits strong effects in the hippocampus (Ren et al., 2018). The task design for 2nd visit of patients (2 years after the first visit) is the same as for 1st visit with different news clips employed.

To test for the confidence and accuracy of recall of events from the news clips, we undertook a post-fMRI questionnaire task. The participants were presented with a question with two answers (one correct and one incorrect) regarding the content of the news clips. For this task, participants were first shown a scene from a continuing news clip and then they answered a question about this news by selecting between two alternative choices. After each question, participants rated their confidence in their answer with an adjustable sliding bar ranging from 0-100 % corresponding to their confidence about the answers. Two questions were asked for each full news clip i.e. 18 questions randomised for each participant.

C.2 MRI processing

The MPRAGE T1-weighted brain scans are segmented using FreeSurfer 6.0 (Fischl, 2012) and in-house implementation of the expectation maximisation segmentation

(Van Leemput et al., 1999). The MP2RAGE T1-weighted scans are first skull-stripped using the method described in (Haast et al., 2018) and then segmented with the methods used for MPRAGE image segmentation. To account for the systematic bias induced by standard partial volume estimation methods on MP2RAGE cortical thickness and brain structure volume measurements (Duche et al., 2014), the MP2RAGE partial volume is computed with a model based on the use of the MP2RAGE Bloch equations (Duche et al., 2017), and cortical thickness computed using a Lagrangian-Eulerian PDE approach (Acosta et al., 2009).

White matter hyperintensities are automatically quantified from T2-weighted FLAIR images using HyperIntensity Segmentation Tool (HIST), which is implemented based on an ensemble of pre-trained neural network classifier (Manjon et al., 2018).

DWI images are first pre-processed for intra-volume motion removal, correction for head motion, eddy currents and intensity inhomogeneities (Andersson and Sotiropoulos, 2016; Glasser et al., 2013). Fractional anisotropy (FA) images are calculated from diffusion tensor estimation from the pre-processed diffusion data using MRtrix 3.0 (Tournier et al., 2019). Fibre orientation distribution (FOD) is estimated using multi-tissue constrained spherical deconvolution (Jeurissen et al., 2014).

An R2* map is computed using the 9-echo GRE data by a voxel-wise exponential fitting with a non-zero offset. An average QSM map is computed by performing the following operations on each GRE echo: FSL-bet brain mask, Laplacian-based phase unwrapping, VSHARP background field removal (Li et al., 2014; Wu et al., 2012), and dipole inversion using STI-suite v2.2 software (Li et al., 2011).

Abdominal DIXON images are processed for assessing visceral adipose tissue (VAT) volumes within the abdominal region defined by anatomical references of vertebral

bodies L1 and L5. The fat-fraction images, i.e., fat fraction = fat/(fat + water) are first computed and used to estimate the mask of adipose tissues using fuzzy c-means clustering (Roullier et al., 2007). The separation of subcutaneous (SAT) and visceral adipose tissues is performed by graph cuts (Boykov et al., 2001), and the VAT segmentation is post-processed by excluding adipose tissue outside the abdominal skeletal muscles and at the posterior of the spine.

C.3 PET Processing

Amyloid-β plaque depositions are automatically quantified from the [^{18}F]FBB PET image using CapAIBL (https://milxcloud.csiro.au/tools/capaibl), a PET-only approach (Bourgeat et al., 2018; Bourgeat et al., 2015). In brief, an adaptive atlas is automatically fitted to each PET image to match its PET retention pattern. Each PET image is spatially normalised to the best fitting atlas, and rescaled using the standardised uptake value ratio (SUVR) by dividing its uptake value to that of the cerebellum cortex. Neocortical [^{18}F]FBB tracer retention is estimated as the average SUVR of the area-weighted mean of frontal, superior parietal, lateral temporal and anterior and posterior cingulate regions of the brain. A PET quantification report is generated from CapAIBL, which illustrates the [^{18}F]FBB SUVR map on the brain surface and indicates the level of amyloid burden.

D. Baseline clinical pathology tests for PISA Onsite

Panel	Full blood count	Biochemistry Panel, CRP, Chols , Trigs, HDL	Serum Folate, Vitamin B12	Iron Studies, Iron binding studies	Ceruloplasmin Protein	Serum Butyrylcholinesterase
Tests	Routine Haematology<br>Haemoglobin<br>Whole Cell Count<br>Platelets<br>Haematocrit<br>MCH<br>Red Cell Count<br>MCV<br>Neutrophils<br>Lymphocytes<br>Monocytes<br>Eosinophils<br>Basophils	Routine chemical pathology<br>Sodium<br>Potassium<br>Chloride<br>Bicarbonate<br>Anion Gap<br>Glucose<br>Urea<br>Creatinine<br>Urea/Creat<br>GFR(estimated)<br>Urate<br>Protein (Total)<br>Albumin<br>Globulim<br>Bilirubin (Total)<br>Bilirubin (Conj)<br>Alkaline Phosphatase<br>Gamma-GT<br>Alanine Transaminase<br>Aspartate Transaminase<br>Lactate Dehydrogenase<br>Calcium<br>Calcium (Alb. Corr.)<br>Phosphate<br>Magnesium<br>Osmolality (Calculated)<br><br>Lipids Chemical Pathology	Megaloblastics Haematology<br>Serum Folate, Vitamin B12	Iron Studies chemical pathology<br>Iron<br>Trasferrin Binding Capacity<br>Transferrin<br>Trasnferrin Saturation<br>Ferritin<br>CRP<br>Creatinine<br>ALT	Trace & Toxic Elements chemical pathology<br>Copper (Serum)<br>Ceruloplasmin<br>Cerulopasmin<br>Cu/Ceruloplasmin Ratio.	Cholinesterase Studies Chemical Pathology<br>Cholinesterase (plasma)<br>Dibucaine number<br>Fluoride number

		Cholesterol<br>Triglyceride<br>HDL Chol.<br>Total/HDL Chol ratio<br>LDL Chol. (Calc)<br>VLDL Chol. (Calc)<br><br>Proteins Chemical Pathology<br>CRP				

References

Acosta, O., Bourgeat, P., Zuluaga, M.A., Fripp, J., Salvado, O., Ourselin, S., Alzheimer's Disease Neuroimaging, I., 2009. Automated voxel-based 3D cortical thickness measurement in a combined Lagrangian-Eulerian PDE approach using partial volume maps. Med Image Anal 13(5), 730-743.

Andersson, J.L.R., Sotiropoulos, S.N., 2016. An integrated approach to correction for off-resonance effects and subject movement in diffusion MR imaging. Neuroimage 125, 1063-1078.

Bourgeat, P., Dore, V., Fripp, J., Ames, D., Masters, C.L., Salvado, O., Villemagne, V.L., Rowe, C.C., group, A.r., 2018. Implementing the centiloid transformation for (11)C-PiB and beta-amyloid (18)F-PET tracers using CapAIBL. Neuroimage 183, 387-393.

Bourgeat, P., Villemagne, V.L., Dore, V., Brown, B., Macaulay, S.L., Martins, R., Masters, C.L., Ames, D., Ellis, K., Rowe, C.C., Salvado, O., Fripp, J., Group, A.R., 2015. Comparison of MR-less PiB SUVR quantification methods. Neurobiol Aging 36 Suppl 1, S159-166.

Boykov, Y., Veksler, O., Zabih, R., 2001. Fast Approximate Energy Minimization via Graph Cuts. IEEE Trans. Pattern Anal. Mach. Intell. 23(11), 1222–1239.

Duche, Q., Raniga, P., Egan, G.F., Acosta, O., Gambarota, G., Salvado, O., Saint-Jalmes, H., 2014. New partial volume estimation methods for MRI MP2RAGE. Med Image Comput Comput Assist Interv 17(Pt 3), 129-136.

Duche, Q., Saint-Jalmes, H., Acosta, O., Raniga, P., Bourgeat, P., Dore, V., Egan, G.F., Salvado, O., 2017. Partial volume model for brain MRI scan using MP2RAGE. Human brain mapping 38(10), 5115-5127.

Fischl, B., 2012. FreeSurfer. Neuroimage 62(2), 774-781.

Glasser, M.F., Sotiropoulos, S.N., Wilson, J.A., Coalson, T.S., Fischl, B., Andersson, J.L., Xu, J., Jbabdi, S., Webster, M., Polimeni, J.R., Van Essen, D.C., Jenkinson, M., Consortium, W.U.-M.H., 2013. The minimal preprocessing pipelines for the Human Connectome Project. Neuroimage 80, 105-124.

Haast, R.A.M., Ivanov, D., Uludag, K., 2018. The impact of B1+ correction on MP2RAGE cortical T1 and apparent cortical thickness at 7T. Human brain mapping 39(6), 2412-2425.

Jeurissen, B., Tournier, J.D., Dhollander, T., Connelly, A., Sijbers, J., 2014. Multi-tissue constrained spherical deconvolution for improved analysis of multi-shell diffusion MRI data. Neuroimage 103, 411-426.

Lambert, J.C., Ibrahim-Verbaas, C.A., Harold, D., Naj, A.C., Sims, R., Bellenguez, C., Jun, G., Destefano, A.L., Bis, J.C., Beecham, G.W., Grenier-Boley, B., Russo, G., Thornton-Wells, T.A., Jones, N., Smith, A.V., Chouraki, V., Thomas, C., Ikram, M.A., Zelenika, D., Vardarajan, B.N., Kamatani, Y., Lin, C.F., Gerrish, A., Schmidt, H., Kunkle, B., Dunstan, M.L., Ruiz, A., Bihoreau, M.T., Choi, S.H., Reitz, C., Pasquier, F., Hollingworth, P., Ramirez, A., Hanon, O., Fitzpatrick, A.L., Buxbaum, J.D., Campion, D., Crane, P.K., Baldwin, C., Becker, T., Gudnason, V., Cruchaga, C., Craig, D., Amin, N., Berr, C., Lopez, O.L., De Jager, P.L., Deramecourt, V., Johnston, J.A., Evans, D., Lovestone, S., Letenneur, L., Moron, F.J., Rubinsztein, D.C., Eiriksdottir, G., Sleegers, K., Goate, A.M., Fievet, N., Huentelman, M.J., Gill, M., Brown, K., Kamboh, M.I., Keller, L., Barberger-Gateau, P., McGuinness, B., Larson, E.B., Green, R., Myers, A.J., Dufouil, C., Todd, S., Wallon, D., Love, S., Rogaeva, E., Gallacher, J., St George-Hyslop, P., Clarimon, J., Lleo, A., Bayer, A., Tsuang, D.W., Yu, L., Tsolaki, M., Bossu, P., Spalletta, G., Proitsi, P., Collinge, J., Sorbi, S., Sanchez-Garcia, F., Fox,

N.C., Hardy, J., Naranjo, M.C., Bosco, P., Clarke, R., Brayne, C., Galimberti, D., Mancuso, M., Matthews, F., European Alzheimer's Disease, I., Genetic, Environmental Risk in Alzheimer's, D., Alzheimer's Disease Genetic, C., Cohorts for, H., Aging Research in Genomic, E., Moebus, S., Mecocci, P., Del Zompo, M., Maier, W., Hampel, H., Pilotto, A., Bullido, M., Panza, F., Caffarra, P., Nacmias, B., Gilbert, J.R., Mayhaus, M., Lannfelt, L., Hakonarson, H., Pichler, S., Carrasquillo, M.M., Ingelsson, M., Beekly, D., Alvarez, V., Zou, F., Valladares, O., Younkin, S.G., Coto, E., Hamilton-Nelson, K.L., Gu, W., Razquin, C., Pastor, P., Mateo, I., Owen, M.J., Faber, K.M., Jonsson, P.V., Combarros, O., O'Donovan, M.C., Cantwell, L.B., Soininen, H., Blacker, D., Mead, S., Mosley, T.H., Jr., Bennett, D.A., Harris, T.B., Fratiglioni, L., Holmes, C., de Bruijn, R.F., Passmore, P., Montine, T.J., Bettens, K., Rotter, J.I., Brice, A., Morgan, K., Foroud, T.M., Kukull, W.A., Hannequin, D., Powell, J.F., Nalls, M.A., Ritchie, K., Lunetta, K.L., Kauwe, J.S., Boerwinkle, E., Riemenschneider, M., Boada, M., Hiltunen, M., Martin, E.R., Schmidt, R., Rujescu, D., Wang, L.S., Dartigues, J.F., Mayeux, R., Tzourio, C., Hofman, A., Nothen, M.M., Graff, C., Psaty, B.M., Jones, L., Haines, J.L., Holmans, P.A., Lathrop, M., Pericak-Vance, M.A., Launer, L.J., Farrer, L.A., van Duijn, C.M., Van Broeckhoven, C., Moskvina, V., Seshadri, S., Williams, J., Schellenberg, G.D., Amouyel, P., 2013. Meta-analysis of 74,046 individuals identifies 11 new susceptibility loci for Alzheimer’s disease. Nat Genet 45(12), 1452-1458.

Li, W., Avram, A.V., Wu, B., Xiao, X., Liu, C., 2014. Integrated Laplacian-based phase unwrapping and background phase removal for quantitative susceptibility mapping. NMR in biomedicine 27(2), 219-227.

Li, W., Wu, B., Liu, C., 2011. Quantitative susceptibility mapping of human brain reflects spatial variation in tissue composition. Neuroimage 55(4), 1645-1656.

Manjon, J.V., Coupe, P., Raniga, P., Xia, Y., Desmond, P., Fripp, J., Salvado, O., 2018. MRI white matter lesion segmentation using an ensemble of neural networks and overcomplete patch-based voting. Comput Med Imaging Graph 69, 43-51.

Ren, Y., Nguyen, V.T., Sonkusare, S., Lv, J., Pang, T., Guo, L., Eickhoff, S.B., Breakspear, M., Guo, C.C., 2018. Effective connectivity of the anterior hippocampus predicts recollection confidence during natural memory retrieval. Nature communications 9(1), 4875.

Roullier, V., Cavaro-Ménard, C., Calmon, G., Aubé, C., 2007. Fuzzy algorithms: Application to adipose tissue quantification on MR images. Biomedical Signal Processing and Control 2(3), 239-247.

Tournier, J.D., Smith, R., Raffelt, D., Tabbara, R., Dhollander, T., Pietsch, M., Christiaens, D., Jeurissen, B., Yeh, C.H., Connelly, A., 2019. MRtrix3: A fast, flexible and open software framework for medical image processing and visualisation. Neuroimage 202, 116137.

Van Leemput, K., Maes, F., Vandermeulen, D., Suetens, P., 1999. Automated model-based tissue classification of MR images of the brain. IEEE Trans Med Imaging 18(10), 897-908.

Wu, B., Li, W., Guidon, A., Liu, C., 2012. Whole brain susceptibility mapping using compressed sensing. Magnetic resonance in medicine 67(1), 137-147.
